# Adoptive cell therapy with CD4^+^ T helper 1 cells and CD8^+^ cytotoxic T cells enhances complete rejection of an established tumour, leading to generation of endogenous memory responses to non-targeted tumour epitopes

**DOI:** 10.1038/cti.2017.37

**Published:** 2017-10-20

**Authors:** Kunyu Li, Braeden Donaldson, Vivienne Young, Vernon Ward, Christopher Jackson, Margaret Baird, Sarah Young

**Affiliations:** 1 Department of Pathology, Dunedin School of Medicine, University of Otago, Dunedin, New Zealand; 2 Department of Microbiology and Immunology, Otago School of Medical Sciences, University of Otago, Dunedin, New Zealand; 3 Department of Medicine, Dunedin School of Medicine, University of Otago, Dunedin, New Zealand

## Abstract

The results of adoptive T-cell therapies (ACTs) are very encouraging and show clinical evidence that ACT can provide a cure for patients with metastatic disease. However, various response rates and long-term cancer remission have been observed in different ACT trials. The types of T cells, prior treatment with chemotherapy and co-administration of other immune-target therapies have been found to influence the efficacy of ACT. In this study, we investigate the ability of ACT using CD4^+^ T helper 1 (Th1) cells and CD8^+^ cytotoxic T lymphocytes (CTLs) to reject the growth of established B16-ovalbumin (OVA) melanoma. CD8^+^ CTLs were found to be the main effector T cells that mediated tumour regression. However, low tumour-free survival rates were observed in ACT with CD8^+^ CTLs only. Co-transferring CD4^+^ Th1 cells and CD8^+^ CTLs has been observed to induce a synergistic antitumour response, resulting in complete regression in 80% of the tumour-bearing mice. We also examined a prior Dacarbazine (DTIC) and after virus-like particle (VLP)-OVA vaccine treatment to enhance ACT, but no therapeutic benefit was observed during primary B16-OVA tumour growth. Nevertheless, the ACT-mediated antitumour response was able to generate memory responses to both B16-OVA and B16-gp33 tumours. VLP-OVA vaccination following ACT enhances the memory responses to tumours that express a heterogenic population of both B16-OVA and B16-gp33 cells; however, it abolished the memory response to tumours consisting of only gp33-expressing cells. These findings provide important information for designing therapeutic treatments for patients with metastatic disease and cancer relapse to achieve durable cancer remission.

Adoptive T-cell therapy (ACT) has become an increasingly attractive modality for the treatment of cancer, due to its high specificity and promise of long-term immune-protection. In particular, it has been suggested as a clinical path to a more effective cancer treatment for patients with metastatic disease.^1^ ACT employs the technique whereby tumour-reactive T cells are infused back into the cancer patient after being stimulated and expanded *ex vivo*. This treatment strategy has shown an objective clinical response rate between 40% and 70% in patients who received a prior lymphodepletion and systemic interleukin (IL)-2 administration.^2, 3, 4^ The majority of these ACT products comprise of CD8^+^ cytotoxic T lymphocytes (CTLs), due to their great expansion capacity. Large numbers of T cells are often used for ACT, and therefore, prolonged cell expansion is required to obtain desirable numbers. Availability of cytokines such as IL-2 affects the proliferation, viability and antitumour reactivity of the adoptively transferred T cells. As a consequence, systemic IL-2 administration was often given to patients after CD8^+^ CTL infusion.^5, 6, 7^ However, this has led to significant side effects, such as capillary leakage.^8^

The findings of ACT in clinical trials and animal studies suggest that the differentiation state of the adoptively transferred cells, the use of lymphodepletion and strategies to boost T-cell proliferation after adoptive transfer influence the efficacy of ACT.^9, 10, 11, 12^ Previously, we have shown that CD4^+^ T helper 1 (Th1) cells were able to enhance CD8^+^ CTL-mediated tumour rejection without systemic administration of recombinant IL-2.^13^ The differentiation state is an important determinant for the longevity of the adoptively transferred T cells. Prior chemotherapy has also been given to patients to facilitate engraftment and homeostatic proliferation of the adoptively transferred T cells.^14, 15^ Dacarbazine (DTIC) is one of the approved alkylating antineoplastic agents that works by adding an alkyl group to the DNA of cancer cells, resulting in DNA damage and cell cycle arrest. It has been administered as a first-line treatment for metastatic melanoma since the 1970s.^16, 17^ DTIC has also been shown to inhibit murine melanoma cell growth *in vitro* and synergise with other antitumour treatments to delay tumour growth in animal cancer models.^18, 19, 20^ Another factor that affects T-cell proliferation after transfer is the poor immunogenicity of tumour cells. A further approach to enhance proliferation of antigen-specific T cells is through vaccination. Vaccination with tumour-associated antigens (TAAs) has been reported to lead to expansion and accumulation of CD8^+^ CTLs within the tumour, resulting in enhancement of tumour regression.^21, 22^ Previously, we have reported that virus-like particles (VLP) derived from rabbit haemorrhagic virus (RHDV) can be used as a vaccine construct to deliver TAAs to elicit a proliferative response of antigen-specific T cells and subsequent elimination of target cells *in vivo*.^23^ In addition, we have also shown that TAA-expressing RHDV VLP were able to inhibit tumour growth in both a therapeutic and prophylactic manner.^24^ In this study, we investigated the efficacy of ACT with T cells that were expanded for different periods of time to mediate remission of established tumours, using a murine model of melanoma. Prior chemotherapy with DTIC and after vaccination with a VLP vaccine expressing the model TAA, ovalbumin (OVA), was also examined to enhance the responsiveness of ACT.

Tumour relapse represents a major clinical challenge. Constant mutation of the tumour cells and loss of TAAs allows tumour cells to evade T-cell recognition.^25^ This results in recurrence of a secondary tumour to which the immune system is tolerant. Induction of antigen-specific immune responses to multiple tumour antigens or mutated antigens is necessary to target tumour recurrence. As such, we also examined the generation of immune memory to secondary tumours that express the ACT target and non-target tumour epitopes in this study.

## Results

### Less differentiated CD4^+^ T cells are more effective in augmenting CD8^+^ T-cell-mediated antitumour immune responses

**Figure 1:**
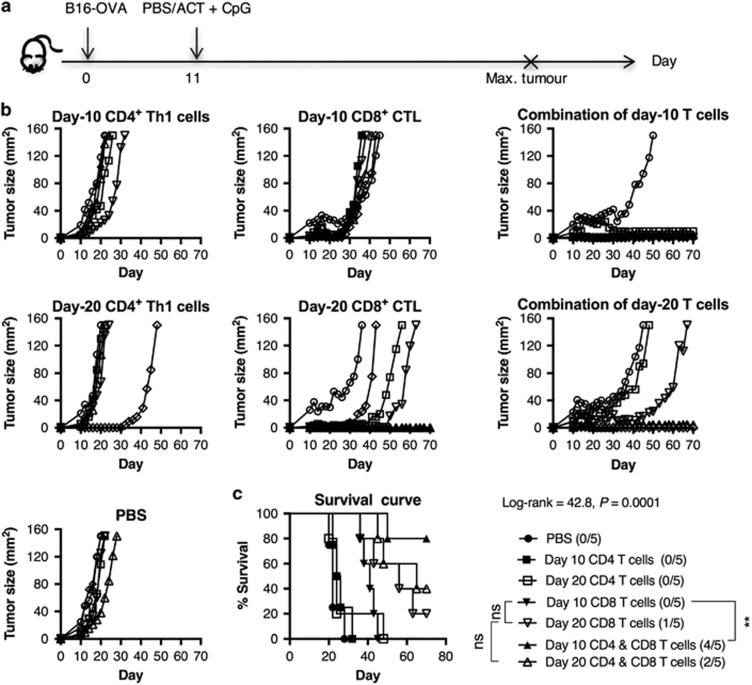
CD4 Th cells expanded for a shorter period of time are more capable of enhancing CD8 CTL antitumour response. Naive C57BL/6 mice were (s.c.) injected with 5 × 10^4^ B16-OVA cells on day 0 and randomised into seven different groups (*n*=5) when the tumours became palpable. On day 11, the mice were (i.v.) injected with PBS or 5 × 10^6^ 10 or day-20 *in vitro* expanded CD4^+^ OT-II cells and/or CD8^+^ OT-I cells alone or in combination. CpG (20 μg per mouse) were given s.c. on day 11 (**a**). Tumour growth was monitored and the mice were killed once tumour size reached 150 mm^2^; (**b**) tumour growth curve and (**c**) survival curve. Statistical analysis was performed with Log-rank (Mantel–Cox) test for survival and one-way analysis of variance to compare survival between treatments with CD8^+^ CTL^+/−^ CD4^+^ Th1 cells. *P*-value<0.05 was considered to be statistically significant; **P<0.01.

The antitumour function of T cells is closely associated with their differentiation state. We were intrigued to find out whether differential tumour responses would be observed in ACT using CD4^+^ Th1 cells and/or CD8^+^ CTLs that had been cultured for different periods of time. Therefore, B16-OVA tumour-bearing mice were treated with 5 × 10^5^ of each day-10 or day-20 *in vitro* expanded CD4^+^ Th1 cells and/or CD8^+^ CTLs (Figure 1a). For CD4^+^ T cells, approximately 60–200-fold cell expansion was obtained, while approximately 500-fold expansion was observed in CD8 T cells, after both primary and secondary expansions (Supplementary Figure S1a). As shown in Figures 1b and c, delay of tumour growth mainly occurred in mice receiving an ACT product containing CD8^+^ CTLs. Single-cell therapy with day-10 CD8^+^ CTLs moderately suppressed tumour growth without inducing tumour-free survival; whereas treatment with day-20 cells resulted in complete tumour regression in 20% of the B16-OVA-bearing mice (Figure 1c). Co-transferring day-20 CD4^+^ Th1 cells led to complete tumour remission in 40% of the mice (Figure 1c). By contrast, a combination of day-10 CD4^+^ Th1 cells and CD8^+^ CTLs resulted in significantly higher tumour-free survival rate of 80% compared with that of day-10 CD8^+^ CTLs alone (Figure 1c). These observations indicate that CD8^+^ CTL represent the main effector cells that inhibit tumour growth. However, coordination of less differentiated CD4^+^ Th1 cells and CD8^+^ CTLs is important for the induction of complete tumour regression.

### Less differentiated CD4^+^ T cells have a greater proliferation capacity *in vivo*

**Figure 2:**
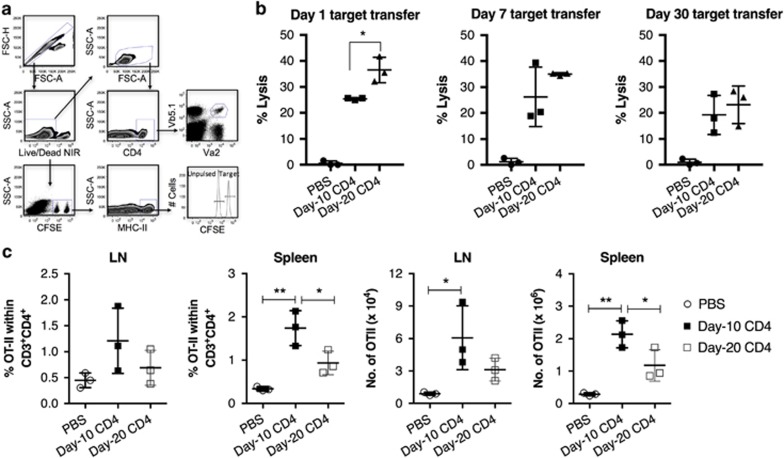
CD4^+^ Th1 cells that expanded for a short and extended period of time have a similar persistence after adoptive transfer. Naive C57BL/6 mice (*n*=3 per group) were (i.v.) injected with DPBS or 2 × 10^6^ day 10 or day 20 *in vitro* expanded CD4^+^ OT-II cells on day 0. Donor splenocytes consisting of equal numbers of OVA_323–339_-pulsed/CFSE^hi^ and unpulsed/CFSE^lo^ cells were (i.v.) injected into the recipient mice 24 h, 7 days or 30 days after T-cell infusion. The mice were sacrificed 40 hr post target cell injection; specific lysis of MHC-II-restricted cells in the lymph nodes and spleens were isolated for flow cytometric analysis. The number of CD4^+^ OT-II T cells in the LN and spleen of mice from the cytotoxicity assay were counted using a haemocytometer and trypan blue to exclude dead cells. The cells from these tissues were stained with Live/Dead dye and the surface markers CD3, CD4, Vα2 and Vβ5.1/5.2 to examine the presence of OT-II cells by flow cytometric analysis (**a**); percentage lysis (**b**); (**c**) percentage and total number of CD4^+^ OT-II cells. The results shown are a representative of two independent experiments with similar trends, error bars=s.e.m. Statistical analysis was performed with one-way analysis of variance to compare mice that received either PBS or CD4^+^ Th1 cell transfer; *P<0.05, **P<0.01.

One of the key factors associated with ACT to induce a durable clinical response is the persistence of tumour-reactive T cells following T-cell infusion.^26^ Previously, we observed a differential capacity of day-10 and day-20 CD4^+^ Th1 cells to enhance the antitumour response of CD8^+^ CTLs. To find out whether this observation was associated with the survival of CD4^+^ Th1 cells, we examined their persistence after adoptive transfer. This was determined by lysis of major histocompatibility complex class II (MHC-II)-restricted OVA_323–339_-expressing cells that were injected into mice at different time points after CD4^+^ Th1 cell infusion (Figure 2a). Specific lysis of target cells were observed at all time points in mice receiving either day-10 or day-20 CD4^+^ Th1 cells (Figure 2b), indicating similar survival of both T cells after transfer. However, higher levels of OT-II cells observed in the spleen of mice receiving day-10 rather than day-20 T cells suggests a greater *in vivo* proliferation capacity of the less differentiated cells (Figure 2c).

### Combination therapies of DTIC, ACT and VLP-OVA vaccine does not enhance tumour regression

**Figure 3:**
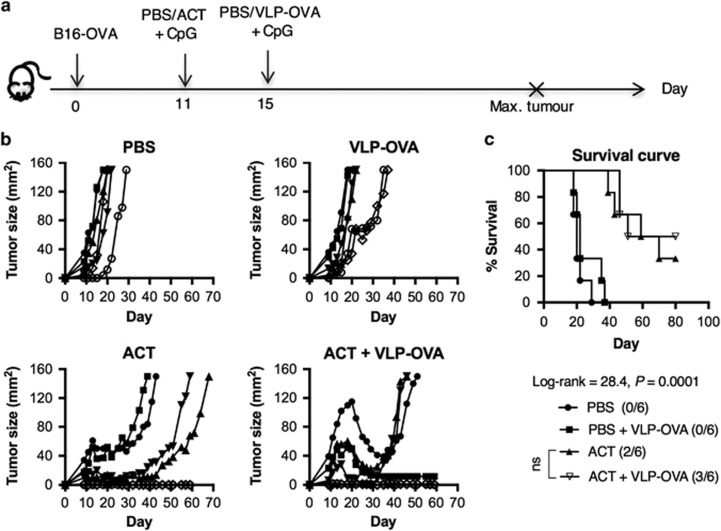
ACT with or without VLP-OVA vaccine treatment induces similar inhibition of B16-OVA tumour. Naive C57BL/6 mice were (s.c.) injected with 5 × 10^4^ B16-OVA cells on day 0 and randomised into 4 different groups (*n*=6) when the tumours became palpable. On day 11, the mice were treated with either PBS or 2.5 × 10^6^ each of day-10 *in vitro* expanded CD4^+^ OT-II cells and CD8^+^ OT-I cells (i.v.) in combination. The mice were vaccinated with PBS or VLP-OVA (100 μg per mouse) (s.c.) on day 15 (**a**). CpG (20 μg per mouse) was given (s.c.) as adjuvant along with both ACT and vaccination. Tumour growth was monitored and the mice were killed once tumour size reached 150 mm^2^; (**b**) tumour growth curve and (**c**) survival curve. Statistical analysis was performed with Log-rank (Mantel–Cox) test for survival. One-way analysis of variance to compare treatment with ACT^+/−^ VLP-OVA vaccine.

**Figure 4:**
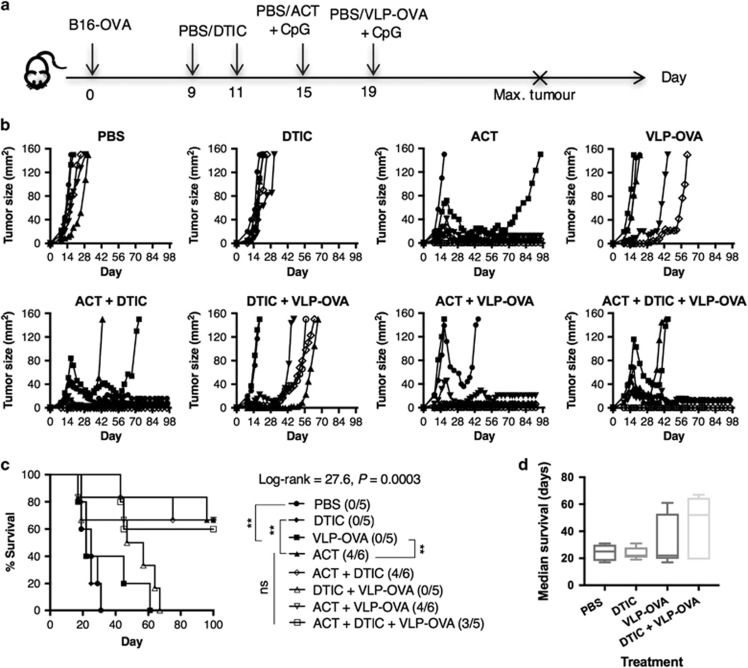
ACT with or without DTIC and VLP-OVA vaccine treatment induce similar rejection of primary tumours. Naive C57BL/6 mice were (s.c) injected with 5 × 10^4^ B16-OVA cells on day 0 and randomised into 8 different groups (*n*=5–6) when the tumours became palpable. The mice were treated with (i.p) PBS or 100 mg kg^−1^ DTIC on days 9 and 11; (i.v) PBS or 2.5 × 10^6^ each of day-10 *in vitro* expanded CD4^+^ OT-II cells and CD8^+^ OT-I cells+(s.c) CpG on day 15; and (s.c) CpG alone or VLP-OVA+CpG in PBS on day 19 (**a**); tumour growth (**b**), survival curve (**c**) and median survival time (**d**). Statistical analysis was performed with Log-rank (Mantel–Cox) test for survival. One-way analysis of variance to compare survival in different treatment groups, no statistical significance between different ACT treatment groups. NS, not significant.

In clinical investigations of ACT, the desired number of T cells cannot always be obtained through *ex vivo* cell expansion. Therefore, we wished to determine whether addition of an antitumour vaccination could synergise with lower T-cell numbers in ACT to mediate an effective antitumour response. As such, 2.5 × 10^5^ of each day-10 CD4^+^ Th1 cells and CD8^+^ CTLs were given in the combination therapies of ACT and VLP-OVA vaccine (Figure 3a). Owing to the overwhelming mass of B16-OVA tumours, a single treatment with VLP-OVA on day 15 induced only slightly delayed tumour growth but no tumour-free survival was observed. (Figures 3b and c). Complete tumour regression was observed in 33% of the mice treated with ACT alone and in 50% of the mice receiving a combination treatment of ACT and VLP-OVA vaccine (Figure 3c). Next, we evaluated the benefit of chemo-immunotherapy with DTIC and ACT. Despite moderate decline of leukocyte numbers on day 4 after treatment with 100 mg kg^−1^ DTIC (Supplementary Figure S2), similar tumour-free survival rates were observed in all mice that received ACT with or without prior DTIC (Figure 4c). Interestingly, a combination treatment of DTIC and VLP-OVA shows a trend of increased median survival time (52 days) compared with treatment with VLP-OVA only (22 days) (Figure 4d).

### Persistence of CD4^+^ Th1 and CD8^+^ CTL after clearance of tumours

**Figure 5:**
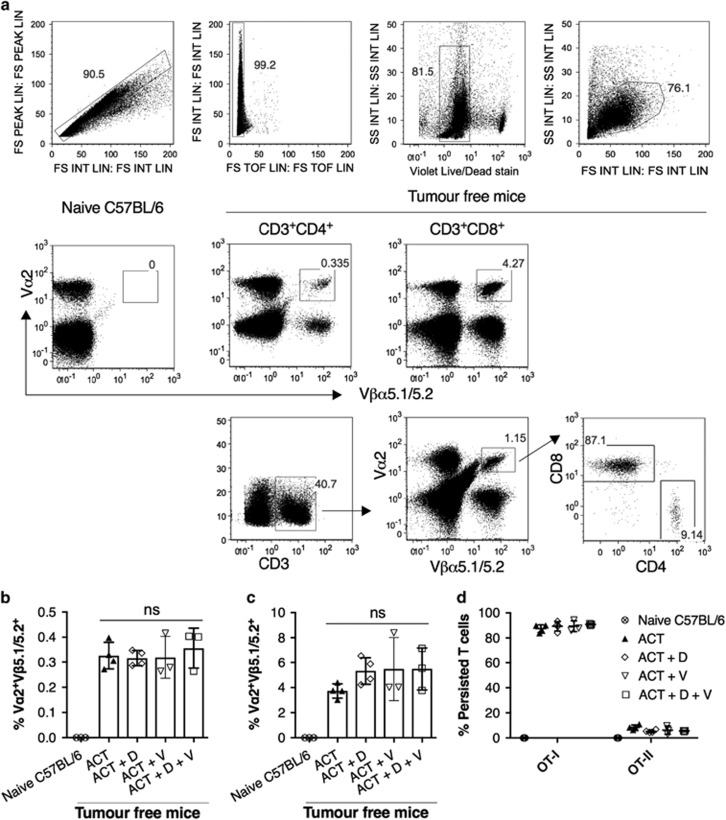
Persistence of CD4 and CD8 CTL after clearance of tumours. Mice that remained free from the primary tumour challenge were tail bled, and the presence of OT-I and OT-II cells in the peripheral blood were examined by flow cytometry; (**a**) gating strategy, (**b**) percentage of OT-II cells within the CD3^+^CD4^+^ compartment, (**c**) percentage of OT-I cells within the of CD3^+^CD8^+^ compartment, (**d**) proportion of OT-I and OT-II in the peripheral blood, error bars=s.e.m. Statistical analysis was carried out by one-way analysis of variance; no statistical significance was observed. D: DITC; V: VLP-OVA.

Persistence of adoptively transferred T cells has been found to be closely associated with the induction of a durable antitumour response.^27^ To assess persistence of adoptively transferred T cells after clearance of tumours, the presence of CD4^+^ OT-II and CD8^+^ OT-I cells in the peripheral blood were identified (Figure 5a). The CD4^+^ OT-II cells that persisted represented approximately 0.2–0.4% of total CD4^+^ T cells (Figure 5b), while CD8^+^ OT-I cells represented approximately 3–8% of total CD8^+^ T cells (Figure 5c). The levels of these two cell subsets were similar in all tumour free mice, regardless of the ACT treatment they received. Interestingly, 90% of the adoptively transferred T cells that persisted in the mice were CD8^+^ OT-I cells, whereas only 10% were CD4^+^ OT-II cells (Figure 5d). A similar distribution was also found in the lymph nodes and spleens of mice that survived a second B16-OVA tumour challenge (Supplementary Figure S3).

### ACT induces endogenous responses to tumours that are devoid of the original target antigen

**Figure 6:**
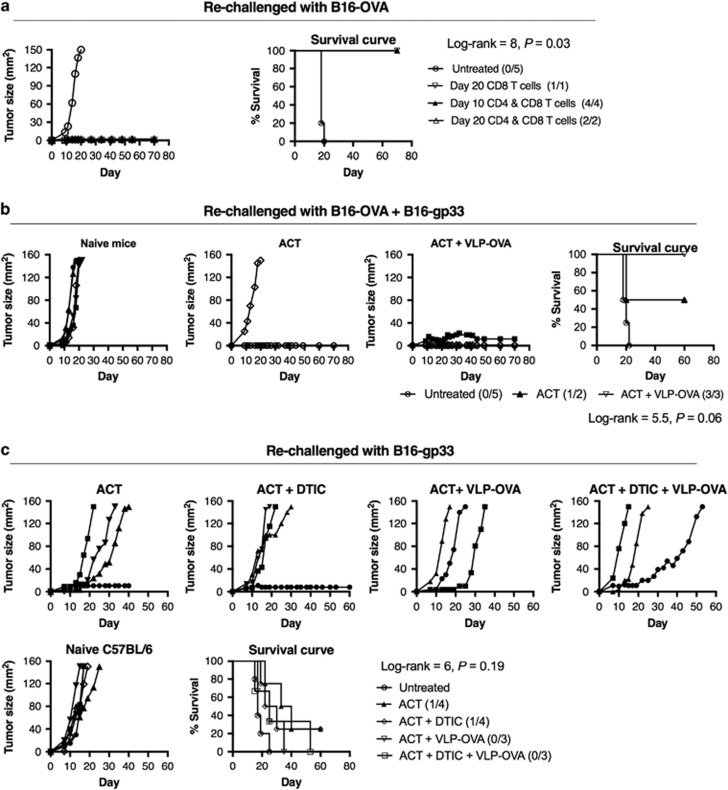
ACT with CD4 Th1-like cells and CD8 CTL induce generation of endogenous immune response to tumour epitopes other than OVA. Naive (*n*=5) and tumour-free mice (from Figures 1,3 and 4) were (s.c.) injected with either 5 × 10^4^ B16-OVA cells (**a**) or total of 5 × 10^4^ B16-OVA and B16-gp33 cells (1:1 ratio) (**b**) or 5 × 10^4^ B16-gp33 cells (**c**). Tumour growth was monitored and the mice were killed once tumour size reached 150 mm^2^; survival in mice received different treatments was compared. Statistical analysis was performed with Log-rank (Mantel–Cox) test for survival.

To determine whether immune memory could be generated after clearance of the primary B16-OVA tumour, mice that remained tumour free 100 days after the primary B16-OVA tumour implantation were re-challenged with a secondary tumour inoculation. Inhibition of the secondary B16-OVA tumour growth occurred in all mice that previously received CD8^+^ CTL, with or without co-transferring CD4^+^ Th1 cells (Figure 6a). To expand upon this finding, we re-challenged the tumour-free mice with a mixture of B16-OVA and B16-gp33 cells to see whether the protection mediated by ACT was effective at rejecting a tumour consisting of a heterogenic population of OVA- and gp33-expressing tumour cells. Regression of secondary tumours was observed in 50% and 100% of the mice that had previously received ACT and a combination of ACT and VLP-OVA vaccine, respectively (Figure 6b). This finding indicates either generation of a stronger endogenous immune memory response to or bystander killing of the B16-gp33 tumours with the addition of VLP-OVA vaccine. To confirm the generation of endogenous T-cell responses to TAAs other than OVA, we inoculated only B16-gp33 cells into mice that had completely rejected the primary B16-OVA tumours. Delay of tumour growth was observed in all mice that survived from the primary B16-OVA tumour, suggesting that T cells specific for tumour epitopes other than OVA were generated. Complete regression of the secondary B16-gp33 tumour occurred in 25% of the mice that previously received ACT with or without DTIC but not in those that received an additional VLP-OVA vaccine (Figure 6c). Nevertheless, these findings suggest that the ACT-mediated antitumour response is able to promote generation of endogenous T-cell response to other tumour epitopes.

## Discussion

ACT has the potential to be developed as a standard cancer treatment; however, there are still several aspects that need to be addressed to determine the key factors associated with a more effective ACT in solid tumours. These include the use of different T-cell subsets, enhancement of *in vivo* persistence of adoptively transferred T cells and induction of endogenous T-cell responses to multiple or mutated tumour epitopes.^26, 28, 29, 30, 31^ Lymphodepleting chemotherapy or total body irradiation are often used together with systemic IL-2 administration to enhance engraftment, survival and antitumour reactivity of the adoptively transferred T cells. Previously, we have shown that ACT using a combination of CD4^+^ Th1 cells and CD8^+^ CTLs induce superior tumour regression than either subset alone. In this study, we investigate the impact of the differentiation state of the T cells in the efficacy of ACT. We demonstrate here that co-transferring less differentiated CD4^+^ Th1 cells and CD8^+^ CTLs without lymphodepletion and IL-2 administration enhances complete tumour remission and also results in generation of an endogenous memory response to non-ACT target epitopes. It is possible that CD4^+^ Th1 cells in the ACT provide a source of IL-2 to support CD8^+^ CTL survival and recruit tumour-specific CD8^+^ CTLs.^32, 33, 34^ CD4^+^ Th cells have also been shown to improve CD8^+^ CTL memory response by retained CD27 expression during lymphocytic choriomeningitis virus infection.^35^

Although earlier attempts at ACT aimed to generate large numbers of T cells, it is still a debate as to whether a high number of more differentiated cells or a lower number of less differentiated cells induce a better therapeutic effect. The disadvantage of using high numbers of T cells in the clinical setting are the labour-intensive T-cell production, and also a very high drop-out rate of enrolled patients due to disease progression or inability to obtain adequate numbers of T cells. These challenges limit the applicability of this treatment approach. Indeed, a study carried out by Wang *et al.*^36^ observed a greater antitumour response in mice receiving a lower number of T cells and total body irradiation. Therefore, generating large numbers of T cells through prolonged *ex vivo* expansion may not be necessary for therapeutic efficacy of ACT. In this study, we examine ACT using both CD4^+^ and CD8^+^ T cells that were expanded for either a short period of 10 days or an extended period of 20 days. We have shown that less differentiated day-10 CD4^+^ Th1 cells and CD8^+^ CTLs induce a greater synergistic antitumour response than that of day-20 cells. Based on the findings of other ACT studies, we examined the phenotype of these *in vitro* expanded T cells for the expression of several markers associated with T-cell differentiation. These include CD27, CD28, CTLA-4, PD-1, CD44, CCR7, CD62L and CD127. Downregulation of CD62L expression from day 10 to day 20 was consistently observed on both CD4^+^ and CD8^+^ T cells (Supplementary Figure S1b). Another mouse study has shown that ACT with antigen-specific CD8^+^ T cells that express the homing receptor CD62L provide a greater antitumour efficacy than ACT using cells that lack CD62L expression.^37^ In addition, we know from our previous study that day-20 cells go through approximately seven more cell cycles than day-10 cells. We have also observed greater proliferation of day-10 CD4^+^ Th1 cells after adoptive transfer (Figure 2). Therefore, superior antitumour responses of less differentiated day-10 T cells may be due to CD62L expression as well as the *in vivo* proliferation capacity of these cells.

Therapeutic regimens that target both the immune system and the tumour have been suggested to be necessary to deliver an effective cancer treatment.^38^ Although DTIC by itself is relatively ineffective in human melanoma, it is still used together with other agents to improve survival of patients with advanced melanoma.^39, 40, 41^ We have examined the benefit of a prior chemotherapy with DTIC to enhance the efficacy of ACT; however, no therapeutic benefit was observed with the dose of DTIC used in this study. The observation of slight reduction in cell numbers with a fast recovery after DTIC treatment may be due the short half-life of DTIC.^42^ Even though the 100 mg kg^−1^ dose used in this study approximates the clinical dose, prolonged DTIC was usually administered in melanoma patients as well as in other animal studies to achieve marked lymphodepletion.^39, 43^ However, this could lead to immunosuppression and enhancement of tumour growth in an aggressive B16 melanoma model. Because of this, we limited the administration of DTIC to two to avoid chemotherapy-associated immunosuppression. Nevertheless, it shows a trend towards better survival when used in combination with VLP-OVA vaccine.

We have also determined the persistence of both CD4 Th1 and CD8 CTLs after clearance of both primary and secondary tumours. Interestingly, although we originally transferred the same numbers of CD4^+^ Th1 cells and CD8^+^ CTLs, most of the cells that persisted after clearance of tumour were CD8^+^ CTLs. It is possible that only a small number of CD4^+^ Th1 cells is required to generate a memory response. One of the important aspects of utilising T cells to kill cancer is having memory function, which allow them to react quickly to the same antigen. Implantation of secondary tumour cells in mice that had completely rejected the primary tumour confirms the generation of memory response to both B16-OVA and B16-gp33 tumour after treatment with ACT. The memory response generated after single therapy ACT is sufficient to prevent engraftment of secondary tumours in the condition of OVA expression. However, it was not sufficient to prevent secondary tumours that express mutated tumour antigens or have lost expression of the originally targeted antigen. From our previous studies, we observed that VLP-OVA vaccination can only effectively control tumour growth when it is given before 7 days after tumour inoculation. Therefore, the observation that VLP-OVA vaccination did not enhance ACT-mediated complete tumour regression may be due to the fact that it was given to tumour-bearing mice on day 15 after tumour inoculation. However, addition of VLP-OVA vaccination shows enhancement of ACT-mediated memory responses to tumours that consist of a heterogeneous population of B16-OVA and B16-gp33 cells. Bystander killing of MHC-II-negative mouse hepatocellular carcinoma and tumour stromal cells have been reported by others.^44, 45^ It is possible that complete rejection of secondary tumours with heterogeneity of antigen expression is due to a combination effect of bystander killing and endogenous response to B16-gp33 cells. However, with VLP-OVA treatment, the strong immunogenicity of OVA antigen could lead to a predominant immune response to OVA-expressing cells, thus weakening the endogenous response to other tumour antigens. This results in poorer immune responses to other tumour epitopes and hence selective growth of variants that do not express OVA. This explains the observation that, when re-challenged with B16-gp33 only, 25% of the mice that were previously treated with ACT^+/−^ DTIC without VLP-OVA rejected engraftment of the secondary tumours, whereas mice that received VLP-OVA vaccination failed to do so (Figure 6c). Vaccination with long tumour peptides that target both the CD4^+^ and CD8^+^ T cells has been found to result in a far more robust antigen-specific T-cell response than that of the short peptide.^46, 47, 48, 49, 50^ Therefore, generation of strong endogenous T-cell responses to multiple tumour epitopes might be desired using vaccine constructs that contain multiple CD4 and CD8 epitopes. We are currently investigating this.

To summarise, this study demonstrated a fascinating antitumour response mediated by the joint action of antigen-specific CD4 Th1-like cells and CD8 CTLs in a mouse model of melanoma. We have shown that a combination of less differentiated CD4^+^ Th1 cells and CD8^+^ CTLs exhibit better antitumour activity than those that have been expanded for a longer period of time. The synergistic response of both cell types seems largely dependent on the differentiation state of the CD4^+^ Th1 cells. Moreover, this ACT-mediated immune response to the primary tumour also led to the generation of endogenous immune response to non-ACT target tumour epitopes. The dose of DTIC used in this study did not benefit the therapeutic response of ACT. However, it shows a trend of better survival with subsequent administration of VLP-OVA vaccine. Perhaps pretreatment of chemotherapy together with an anticancer vaccine may act to reduce the bulk of fast growing tumour prior to ACT.

## Materials and methods

### Animal ethics and approvals

Specific pathogen-free female C57BL/6, OT-I and OT-II mice, 8–12-week old, were sourced from the Hercus Taieri Research Unit (University of Otago, Dunedin, New Zealand). Experimental protocols involving animals were approved by the University of Otago Animal Ethics Committee (Dunedin, New Zealand).

### Antibody staining and flow cytometry

Monoclonal antibodies for flow cytometric analysis were obtained from BD Biosciences (San Jose, CA, USA), Biolegend (San Diego, CA, USA) or eBioscience (San Diego, CA, USA) and carefully titrated prior to use. Cells were first stained with Live/Dead fixable dye (Invitrogen, Grand Island, NY, USA) before surface and/or intracellular staining. Staining of surface antigen was performed in fluorescence-activated cell sorting buffer for 10 min at 4 °C in the dark. All flow cytometric analysis was performed with a BD Fortessa (Becton, Dickinson, Mountain View, CA, USA) or Galios (Beckman Coulter) instrument; and data was analysed with the FlowJo 9.6 software (Ashland, OR, USA).

### Cell culture media recipes

Complete Iscove’s Modified Dulbecco’s Medium (cIMDM-5): IMDM (Gibco, Invitrogen)+1% Penicillin/Streptomycin (Gibco, Invitrogen, San Diego, CA, USA) +0.1% 2-Mercaptoethanal (Gibco, Invitrogen)+5% fetal calf serum advance–Dulbecco’s modified Eagle’s medium (DMEM)/F12 (cA-DMEM/F12-5): advanced-DMEM/F12+ 1% Penicillin/Streptomycin+0.1% 2-mercaptoethanol+1% GlutaMax (Gibco, Invitrogen)+20 mM 4-(2-hydroxyethyl)-1-piperazineethanesulfonic acid solution (Gibco, Invitrogen)+5% fetal calf serum.

### Generation of bone marrow-derived dendritic cells (BMDCs)

C56BL/6 BMDCs were generated as previously described.^51^ Briefly, BM single-cell suspensions were cultured in a six-well plate (Falcon, Corning, NY, USA) at 2.5 × 10^6^ cIMDM-5 supplemented with 20 ng ml^−1^ recombinant granulocyte macrophages colony-stimulating factor (Biosource, ThermoFisher, San Diego, CA, USA) (DC medium). Cultures were fed every 2–3 days by removing 50% of the medium from each well and replenishing with an equal amount of fresh DC medium and incubated at 37 °C/5% CO_2_ for 7 days. For DC maturation, 1 μg ml^−1^ lipopolysaccharide (Sigma Aldrich Co., St Louis, MO, USA) was added to day-6 BMDC cells overnight.

### Isolation of CD4^+^ OT-II and CD8^+^ OT-I cells and MHC-I and MHC-II peptides

Antigen-specific CD4^+^ and CD8^+^ cells were isolated from OT-II and OT-I splenocytes respectively, through magnetic bead separation. Anti-mouse CD4 (clone L3T4) and CD8 (clone Ly-2) microbeads were obtained from Miltenyi Biotec Ltd (Bergisch Gladbach, Germany) and used according to the manufacturer’s instructions. The MHC class I peptide OVA_257–264_ (SIINFEKL) (JPT Peptide, Berlin, Germany) and the MHC class II peptide OVA_323–339_ (ISQAVHAAHAEINEAGR) (Mimotopes Pty Ltd, Notting Hill, VIC, Australia) of OVA were used as the target antigens for CD8^+^ CTL and CD4^+^ Th cells, respectively.

### Expansion of CD4^+^ Th1 cells and CD8^+^ CTLs

Non-adherent and loosely adherent day-7 BMDC cells were harvested, washed and resuspended at 1 × 10^6^ cells ml^−1^ in DC medium. Untreated and lipopolysaccharide-treated BMDCs were then incubated with 1 μg ml^−1^ OVA_323–339_ (DC-OVA_323–339_) or OVA_257–264_ (DC-SIINFEKL) for 4 h at 37 °C/5% CO_2_. Free peptides were washed off once in Dulbecco’s phosphate-buffered saline (DPBS), and the DCs were resuspended in cIMDM-5 at 1 × 10^6^ cells ml^−1^. Naive CD4^+^ OT-II cells and CD8^+^ OT-I cells were stimulated with DC-OVA_323–339_ and DC-SIINFEKL, respectively, and expanded in cA-DMEM/F12-5. The cells were either expanded for a short period of 10 days to generate or for an extended period of 20 days. IL-7 was added at 5 ng ml^−1^ to both cell types throughout the entire period of cell expansion, and 1 ng ml^−1^ IL-2 was added to CD4 Th cells only upon TCR stimulation. For 20 days’ cell expansion, T cells were restimulated with peptide-pulsed DC for 4 h and then separated by gentle pipetting into fresh wells for clonal expansion. Cells were split and fed every 2–3 days to maintain cell concentration at 0.5–1 × 10^6^ cells ml^−1^.

### Preparation of donor splenocytes for cytotoxicity assay

Donor splenocytes from naive C576BL/6 mice were pulsed with OVA_323–339_ or left unpulsed at 5 × 10^6^ cells ml^−1^ in cIMEM-5 for 3 h at 37 °C/5% CO_2_; free peptide was washed off with DPBS three times and the cells were spun down at 350 *g* for 5 min at room temperature. Unpulsed and antigen-pulsed splenocytes were resuspended in DPBS at 2 × 10^7^ cells ml^−1^ and mixed with equal volume of 0.4 μM carboxyfluorescein succinimidyl ester (CFSE) (CFSE^lo^) and 4 μM CFSE (CFSE^hi^), respectively, and incubated for 8 min at room temperature in the dark. An equal volume of fetal calf serum was added to quench the reaction, and the cells were washed three times in DPBS. Unpulsed and peptide-pulsed cells were then mixed at a 1:1 ratio in DPBS at 50 × 10^6^ cells ml^−1^; a total volume of 200 μl was intravenously (i.v.) injected into naive recipient mice after T cell transfer.

### Measurement of CD4^+^ Th1 cell persistence by cytotoxicity assay

On day 0, either PBS or 2 × 10^6^ *in vitro* expanded CD4^+^ OT-II cells were i.v. injected into naive C57BL/6 recipient mice (*n*=6). Donor cells (1 × 10^7^) consisting of equal numbers of OVA_323–339_-pulsed-CFSE^hi^ and unpulsed-CFSE^lo^ were i.v. injected into the recipient mice 24 h, 7 days or 30 days after CD4^+^ OT-II cells’ infusion. The mice were killed 40 h after target cell infusion; lymph nodes and/or spleens were isolated for flow cytometric analysis of target cell lysis. Cells were stained with Live/Dead fixable Near-IR, CD16/32 (2.4G2, BD Biosciences), followed by MHC-II (clone M5/114.15.2, Biolegend). Percentage of lysis of MHC-II^+^ target cells was calculated using the formula: Ratio=number of unpulsed/number of peptide pulsed and percentage of lysis=(1−ratio_absence of E_/ratio_presence of E_) × 100, where E is effector cells.

### Tumour cell lines and tumour trials

B16-OVA and B16-gp33 cells were maintained in cIMDM-5+0.5 mg ml^−1^ Geneticin (Gibco) at 37 °C/5% CO_2_. Tumour cells were harvested from tissue culture flasks by detaching adherent cells with Trypsin/EDTA (Gibco), washed with DPBS and pelleted at 250 *g* for 5 min at room temperature and then resuspended at desired concentration in DPBS for subcutaneous (s.c.) injection into tumour cells in the left flank of the mice. Once the tumour became palpable, the mice were randomised into different groups for different treatments. Tumour growth was monitored every 2 days by measuring the width and length of the tumour with a calliper. Tumour size was calculated as the product of the two tumour bisecting diameters. The mice were killed when the tumours reached 150 mm^2^ size.

### ACT with day-10 and day-20 *in vitro* expanded CD4 Th1 cells and CD8 CTL

B16-OVA-bearing mice (*n*=5) were i.v. injected with DPBS or 5 × 10^6^ day-10 or day-20 *in vitro* expanded CD4^+^ OT-II and CD8^+^ OT-I cells followed by s.c. CpG (20 μg per mouse) injection on day 11 after tumour inoculation.

### Combination of ACT and VLP-OVA vaccine trial

B16-OVA-bearing mice (*n*=6) were i.v. injected with DPBS or 2.5 × 10^6^ day-10 *in vitro* expanded CD4^+^ OT-II and CD8^+^ OT-I cells and s.c. CpG on day 11 after tumour inoculation. RHDV VLP-OVA vaccine was prepared as described previously.^23^ On day 16, mice either received s.c injection of CpG (20 μg per mouse) (sequence:TCCATGACGTTCCTGACGTT, Geneworks, Thebarton, SA, Australia,) alone or VLP-OVA (100 μg per mouse)+CpG in DPBS.

### Combination of DTIC, ACT and vaccine treatment

B16-OVA-bearing mice (*n*=5–6) were intraperitoneally treated with either 0.9% NaCl or 100 mg kg^−1^ DTIC in 0.9% NaCl on days 9 and 11 after tumour inoculation. On day 15, the mice were i.v. injected with either DPBS or 2.5 × 10^6^ day-10 *in vitro* expanded CD4^+^ OT-II and CD8^+^ OT-I cells and s.c. CpG. CpG alone or VLP-OVA+CpG were given s.c. to the mice on day 19.

### Analysis of T-cell persistence after clearance of tumours

Mice that remained tumour free for 100 days after B16-OVA inoculation were tail bled. The presence of OT-I and OT-II cells in the peripheral blood was analysed by flow cytometry with antibodies against mouse CD3 (145.2C11, BD Biosciences), CD4 (GK.15, Biolegend), CD8 (53-6.7, BD Biosciences), Vα2 (B20.1, Biolegend) and Vβ5.1/2 Vα2 (MR9-4, Biolegend).

### Tumour re-challenge

Mice that had completed tumour regression were re-challenged with 5 × 10^4^ B16-OVA, B16-gp33 or a mixture of B16-OVA and B16-gp33 cells in 100 μl DPBS in the left flank. An additional control group of naive C57BL/6 mice (*n*=5) also received s.c. inoculation of the tumour cells in the left flank. Tumour growth was monitored for 70 days.

### Statistical analysis

Statistical analysis was performed with Graph Pad Prism 6 (La Jolla, CA, USA). The particular type of statistical analysis is listed in each relevant figure legend. *P*-value⩽0.05 was considered to be statistically significant.
