# Supplementary material for: Adoptive cell therapy with CD4+ T helper 1 cells and CD8+ cytotoxic T cells enhances complete rejection of an established tumour, leading to generation of endogenous memory responses to non-targeted tumour epitopes
Source: Clin Transl Immunology. 2017 Oct 20;6(10):e160–. doi: 10.1038/cti.2017.37 (PMC5671987; doi:10.1038/cti.2017.37)
Supplement: Supplementary Figures [file cti201737x1.docx]

# Supplementary figures

**Figure S1: Expansion and phenotype analysis of T cells.**

CD4^+^ and CD8^+^ cells isolated from naïve OT-II and OT-I mice, were stimulated with unpulsed DC or DC-OVA_323-339_ and DC-SIINFEKL, respectively. The cells were expanded in A-DMEM/F12-5 for 10 to 20 days. IL-7 was added at 5 ng/ml to both cell types throughout the entire period of cell expansion; and 1 ng/ml IL-2 was added to CD4 Th cells only upon TCR stimulation. Fold of cell expansion was calculated as: Final number of viable cells ÷ Number of viable cells on the day of antigen stimulation. Expression of different maker on both day 10 and day 20 cells was examined by flow cytometric analysis; red line: CD127^-^ (gate on FMO); blue line: CD127^+^

Figure S2: DTIC treatment in B16-OVA bearing mice did not delay tumour growth

Naïve C57BL/6 mice were (s.c) injected with 5 × 10^4^ B16-OVA cells on day 0, and randomised into 5 different groups (n = 9) when the tumours became palpable. The mice were injected (i.p) with PBS, or 25 mg/kg or 100 mg/kg DTIC on day 9 and day 11. Tumour growth was monitored (a), and three mice from each group were sacrificed on day 2, 4, and 6 after the last treatment. Tumour draining, non-draining LN and spleen were isolated, total live cell numbers counted (b).

**Figure S3: Persistence of CD4^+^ Th1 cells and CD8^+^ CTL after clearance of secondary tumours**

Mice that remained tumour free after re-challenge with a mixture of 5 × 10^4^ B16-OVA and B16-gp33 cells, were sacrificed on day 70 post tumour re-challenge. LN and spleen were isolated for analysis of the presence of OT-I and OT-II cells.
